# Preferred reporting items for concept analysis in nursing: a systematic review

**DOI:** 10.1177/17449871251410464

**Published:** 2026-02-04

**Authors:** Abdulqadir J Nashwan, Jibin Kunjavara, George Joy, Kalpana Sigh, Moustaq Karim Khan Rony, Kamarudeen M, Sirwan Khalid Ahmed

**Affiliations:** Nursing and Midwifery Research Department, HMC, Qatar; Nursing and Midwifery Research Department, HMC, Qatar; Nursing and Midwifery Research Department, HMC, Qatar; Nursing and Midwifery Research Department, HMC, Qatar; International University of Business Agriculture and Technology Department of Civil Engineering, Dhaka, Dhaka division Bangladesh; Nursing and Midwifery Research Department, HMC, Qatar; University of Raparin College of Nursing Rania, Sulaymaniyah Governorate, Iraq

**Keywords:** concept analysis, Delphi study, EQUATOR Network, nursing research, PRISMA, reporting guidelines, systematic review

## Abstract

**Background:**

Concepts are fundamental to nursing theory, practice, and research, serving as abstract constructs that represent key aspects of human experience. However, the reporting of concept analysis in nursing lacks standardised guidelines, leading to inconsistencies in methodological transparency and rigour. Existing frameworks for qualitative and mixed-methods research do not adequately address the unique aspects of concept analysis.

**Aims:**

This study aims to develop a comprehensive reporting guideline for concept analysis in nursing research, guided by the EQUATOR Network toolkit.

**Methods:**

The first stage of guideline development involves a systematic review of MEDLINE, CINAHL, and PsycINFO to identify key components of concept analysis reporting in nursing. The review will focus on studies utilising concept analysis within the nursing metaparadigms: person, environment, health, and nursing, while adhering to PRISMA guidelines to ensure methodological rigour.

**Results:**

The systematic review synthesised existing literature to establish a foundational framework of essential elements for reporting concept analysis in nursing.

**Conclusions:**

Findings from this review will inform a subsequent Delphi study and structured guideline development process. The resulting standardised reporting framework will enhance methodological consistency, transparency, and reproducibility in concept analysis, thereby contributing to the advancement of nursing theory, research, and practice.

## Introduction

The rapid evolution of societal value systems has presented nursing with increasing ethical and philosophical challenges in delivering patient care. These shifts have also led to the development of new professional nursing environments to meet emerging healthcare needs(Gunawan et al., 2023). Initially, concept analysis inquiries were limited, and some journals were reluctant to publish such studies. However, over time, these analyses began appearing in various journals, including those with rigorous research methodologies and theoretical peer review. Consequently, ‘concept analysis papers’ have become an integral part of coursework in many graduate nursing programmes (Rodgers et al., 2018).

Concepts are fundamental elements that encompass attributes describing phenomena and offer essential insights for nursing theory development. They provide a fresh perspective for theorists, researchers, and practitioners to observe and explain phenomena of interest within the framework of concepts (Kim, 2023). It is essential to go through a concept analysis phase to define a concept clearly. Conceptual analysis clarifies the meaning of ambiguous concepts by explaining the nature of the phenomena of interest in relation to the specific focus area (Abujaber and Nashwan, 2024). Martínez et al. (2021) analysed reviews assessing the quality of reporting in concept analysis studies, reviewing 31 papers and identifying frequent gaps in critical information across various research phases, in relation to continuity of care. Many papers had overlap with related concepts that were not required for chronic diseases, and there remains confusion and ambiguity regarding the definition of continuity of care in the paper. Authors note that studies frequently exclude essential information about concept analysis (e.g. Hu et al., 2020). This highlights the necessity for standardised guidelines for concept analysis.

A reporting guideline serves as a checklist outlining the essential information that should be included in a manuscript or research report (Enhancing the QUAlity and Transparency Of health Research [EQUATOR] Network, 2021). Numerous studies have demonstrated that adhering to such guidelines enhances the quality, clarity, and transparency of research reports. For instance, a review found that the use of the Preferred Reporting Items for Systematic Reviews and Meta-Analyses (PRISMA) reporting guideline significantly improved the completeness of reporting in over 100 systematic reviews published across five emergency medicine journals (Nawijn et al., 2019).

Furthermore, the lack of available standardised frameworks tailored to specific disciplines underscores the need for discipline‑based concept analysis. In nursing, concepts are deeply embedded in theory, practice, and patient care, and generic analysis methods may not capture the profession’s unique epistemological and contextual requirements (Rodgers et al., 2018). The absence of discipline‑specific guidance promotes inconsistencies in how concept analyses are conducted and reported, limiting comparability and utility (Peavey and Vander Wyst, 2017). Therefore, developing a nursing‑specific framework ensures that conceptual work is both methodologically rigorous and practically relevant within the nursing domain.

No reporting guidelines for concept analysis in nursing research are listed on the EQUATOR network or in other scientific disciplines. Although concept clarification methods are used in various fields such as philosophy, psychology, and the social sciences, there is no standardised framework outlining how such analyses should be reported. We have identified a gap in reporting guidelines specifically tailored for concept analysis. In nursing, concept analysis plays a unique and foundational role in theory development, research instrument construction, and evidence-based practice. For example, concepts such as compassion, comfort, and resilience are central to nursing care, and their clarity directly influences clinical application. However, inconsistencies in how concept analyses are conducted and reported reduce comparability, reproducibility, and academic rigour. Given nursing’s distinct epistemological stance, integrating empirical, theoretical, and experiential knowledge, there is a need for reporting guidance that reflects this complexity. This systematic review aims to identify a reporting item for guideline development of concept analysis in nursing research.

## Research question

What are the most preferred reporting items included in the report of a concept analysis in Nursing?

### Objectives

- Review the quality of reporting of concept analysis studies to establish key areas:
- Where reporting may be sub-optimal.
- To identify the preferred reporting items to include in the report of a concept analysis in nursing.

## Methods

This review complies with the guidelines of the Preferred Reporting Items for Systematic Review and Meta-Analysis Protocols (PRISMA-P) 2020 statement (Page et al., 2021).

### Eligibility criteria

The authors conducted a review that included primary concept analysis papers meeting specific eligibility criteria. The focus was on nursing-related concept analyses published in peer-reviewed journals between 2014 and 2024. The 10-year range was chosen to capture the most contemporary methodological practices and ensure relevance to current nursing theory and research paradigms. Restricting the review to this period provided a manageable and methodologically consistent body of evidence for guideline development.

Only English-language papers were included. Although the exclusion of grey literature may have led to omission of conceptual discussions from dissertations or institutional reports, this decision ensured methodological consistency and peer-reviewed rigour. To minimise potential bias in study selection, we did not conduct backward or forward reference searching. This approach prevented citation bias towards historically prominent studies and enhanced the reproducibility and transparency of the search strategy .To minimise bias in the review methodology, we also did not examine the references of the included studies.

### Information sources

To gather relevant data, we searched three major online database PubMed, Scopus and the Cumulative Index to Nursing and Allied Health Literature (CINAHL) via the Ovid platform. In alignment with the Cochrane Handbook recommendation of using at least three databases for systematic reviews, these sources were selected for their comprehensive coverage of nursing and health science literature, ensuring capture of the majority of relevant concept analysis studies. The initial search was conducted in December 2024. As the objective of this review was not to exhaustively identify all existing concept analysis papers but rather to obtain a representative sample sufficient to identify consistent and preferred reporting items, this targeted approach was considered both methodologically sound and appropriate for the study aim.

### Search strategy

The search strategy for this review involved using the following key terms: For the concept, we included the terms ‘Concept’, ‘Conceptualisation’ and ‘Attribute’. For analysis, we used the terms ‘Analysis’, ‘Structured Analysis’ and ‘Process and Systematic Analysis’. Finally, for nursing, we searched with the terms ‘Nursing services’, ‘Nursing Care’, ‘Nursing Evaluation Research’, ‘Nursing Theory’ and ‘Nurse–Patient relations’. These search terms were combined using Boolean operators to refine the search results.

### Selection process

**Figure 1.**
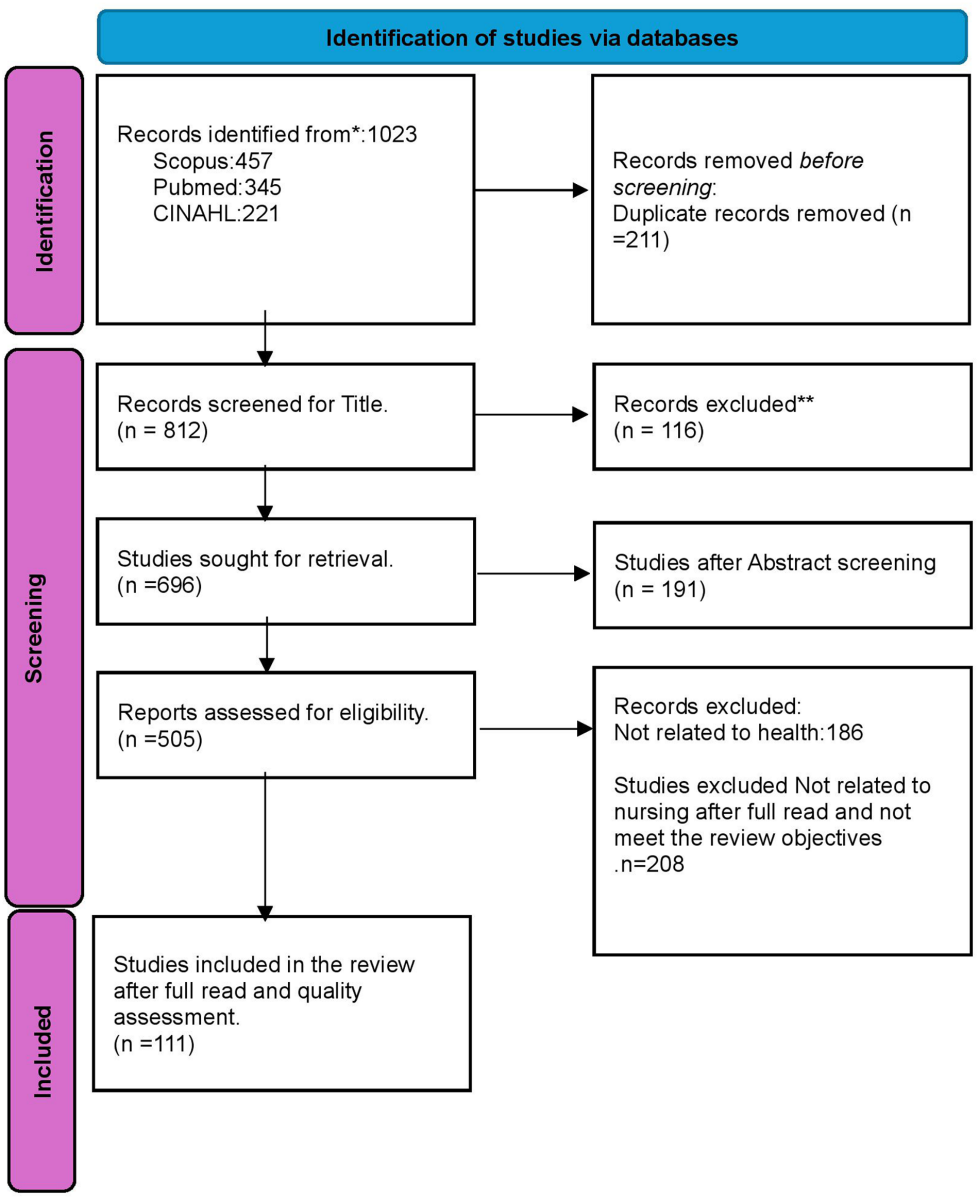
PRISMA flow diagram.

Citations from the three databases were imported into an excel sheet, where duplicates were removed. Two independent reviewers (JK and KM) conducted the title and abstract screening, followed by full-text screening, based on the eligibility criteria. Discrepancies between reviewers were resolved by a third reviewer (GJ). In cases where multiple papers stemmed from the same study, the first reported paper was included, Finally 111 papers were selected for final review. This process is illustrated in the PRISMA diagram below (Figure 1). Due to the volume of papers reviewed a summary table has been included as supplementary material.

### Data collection process

Three reviewers (GJ, KM, and JK) independently extracted data using a structured spreadsheet (Pantha et al., 2023), categorising each item as ‘Reported’, ‘Not Reported’ or ‘Not Applicable’. After reviewing the first ten studies, the team identified that several important elements specific to concept analysis reporting were missing from the original form. To address this, each paper was manually re-examined by the research team to identify additional relevant components, eliminate duplicates and refine unclear items. As a result, 56 new items were added to capture details such as the use of reporting guidelines, methodological rigour, conceptual derivation, and clarity in analytical processes. The final version of the data extraction tool contained 66 items, encompassing both reporting components and study characteristics (Rodgers et al., 2018). The refined list of components was subsequently reviewed by content expert (AJN) who provided feedback that further improved the precision and relevance of each item. All versions of the data extraction forms, illustrating the stepwise refinement process, are included in the Supplemental Materials to ensure methodological transparency and reproducibility.

## Results

Characteristics included in concept analysis were summarised using standard descriptive statistics. We reported the number and percentage of manuscripts that addressed each data item.

This basic dataset on concept analysis presents the distribution of individuals or entities across different geographical regions, with their corresponding numbers and percentages. The majority are from Asia (42%), followed by North America (34%), indicating a strong presence in these two regions. Europe and Australia (14%) make up a smaller portion, whereas Africa (6%) and South America (4%) represent the least share. This distribution highlights the dominance of Asia and North America, suggesting potential regional influences, trends or disparities in participation, engagement, or representation (Table 1).

**Table 1.** Basic characteristics about the included Paper in terms of region.

Geographical region	Number	Percentage
Asia	47	42
North America	38	34
Europe and Australia	15	14
Africa	7	6
South America	4	4

The distribution of published concept analysis papers across nursing specialties indicates a strong emphasis on nursing education (78%), highlighting a predominant scholarly focus on refining educational theories, teaching methodologies, and competency frameworks. In contrast, nursing practice (14%) has fewer publications, suggesting a relatively lower focus on conceptualising clinical care models and intervention strategies. Meanwhile, research and administration (8%) account for the least share, reflecting limited but significant efforts in analysing concepts related to healthcare leadership, policy, and research methodologies. This trend emphasises the priority given to educational advancements in nursing literature, whereas clinical practice and administrative concepts receive comparatively less attention (see Table 2).

**Table 2.** Characteristics of included paper.

Nursing specialties	Number	Percentage
Education	86	78
Practice	16	14
Research and administration	9	8

Table 3 presents data on the number and percentage of publications across three time periods: 2014–2017, 2018–2021 and 2022–2024. It shows the distribution of research output over the years, highlighting an increase in publications between 2018 and 2021, which accounted for the highest proportion (44%). The number of publications then slightly declined in the 2022–2024 period (34%), though it remained significantly higher than in 2014–2017 (19%). This trend suggests a growth in concept analysis productivity over time, with a peak in the 2018–2021 period, followed by a moderate decrease in the latest period.

**Table 3.** Description of papers about the publication year.

Year of publication	Number	Percentage
2014–2017	21	19
2018–2021	49	44
2022–2024	41	34

### Quality of reporting concept analysis study

Supplemental Table 1 presents a total of 66 data extraction items related to the reporting of concept analysis research. These items are categorised under eight key subheadings: (1) Title and Abstract, (2) Background, (3) Methods, (4) Ethics, (5) Results, (6) Discussion, (7) Limitations and (8) Conclusion. The table provides a summary of the components of reporting across the included studies, highlighting the key components of concept analysis in nursing research.

Overall, the review revealed strong reporting in narrative sections such as the Title and Abstract, Background and Discussion, which demonstrated clarity, coherence and relevance (Table 4). However, major weaknesses were evident in the Methods, particularly in the Search, Data and Analysis components, where details were often missing or inconsistently described, limiting transparency and reproducibility. Reporting of Concept Definition, Case Examples, and Antecedents and Consequences varied widely, indicating uneven depth and methodological rigour. Although Empirical Referents and Recommendations were generally well addressed, Ethics considerations were frequently neglected. Overall, the findings highlight the need for greater methodological precision and consistency to enhance the quality and standardisation of concept analysis reporting in nursing research.

**Table 4.** Summary of reporting items in concept analysis studies.

Section	% Reported (Range)	Key note
Title and Abstract	80–96	Strong clarity and reporting
Background/Introduction	88–98	Very well reported
Methods – Search and Framework	41–81	Moderate; search details need improvement
Methods – Data and Analysis	21–57	Weakest area overall
Concept Definition and Attributes	31–91	Variable depth in defining concepts
Case Examples	31–77	Often missing or incomplete
Antecedents and Consequences	46–88	Reported but inconsistent
Empirical Referents	70–86	Good evidence support
Ethics	41	Frequently neglected
Discussion and Implications	76–97	Strong synthesis and relevance
Recommendations and Diagrams	61–70	Present but not universal

#### Title and abstract (Eight items)

The majority of authors, in approximately two-thirds of the papers included explicitly stated in their manuscript titles that they were doing concept analysis research. Nearly all studies offered a scientific rationale for the analysis and provided detailed information about the process within the abstract.

#### Background (Three items)

The majority of studies clearly stated the aim of the research, with over 80% providing a rationale for the concept analysis study design.

#### Methodology (Fourteen items)

Fourteen items including analysis data items were related to the reporting of concept analysis study under six subheadings (which are related to the general category of the Method, Framework, Data Sources and Data Analysis, Discussion and Recommendation). The reporting percentages across different methods highlight variations in transparency and rigour. While framework-based methods show strong reporting in areas like step-by-step methodology (95%), data source methods exhibit moderate reporting, with criteria for selecting sources (63%), and evidence tables (53%) being reasonably documented. However, data analysis methods report lower percentages, particularly in qualitative data synthesis (23%) and analysis techniques (26%). This suggests that while methodological frameworks are well-documented, data collection and analysis require better reporting for comprehensive concept analysis.

#### Results (32 items)

The results highlight key aspects of concept analysis, including concept definition, attributes, cases, antecedents, consequences, and empirical referents. Most studies (91%) discussed the originality of the concept, while over 80% clearly identified key characteristics and attributes. The application of the concept in various settings (65%) and comparisons with similar and contrasting concepts (71%) were also commonly reported. Constructed cases, including model (86%), borderline (77%), and contrary cases (56%), were frequently presented to illustrate concept attributes. Antecedents and consequences were well-defined, with 98% identifying events leading to the concept and 84% discussing personal consequences. Empirical referents (96%) and supporting evidence (78%) were consistently provided. However, hypothesis formulation (18%) and study relevance analysis (34%) were less frequently reported. Ethical considerations (45%) and operational definitions (78%) were also noted, indicating a need for greater emphasis on study justification and ethical discourse in concept analysis research.

## Discussion

This systematic review evaluated current reporting practices in concept analysis studies within nursing research, aiming to guide the development of standardised reporting guidelines. The findings revealed both strengths and inconsistencies across the literature, emphasising the need for a tailored reporting framework specific to concept analysis. Such a framework is crucial to enhance methodological transparency, promote reproducibility, and improve the quality of scholarly outputs in nursing (O’Dea et al., 2021). A notable strength was the generally strong reporting in titles and abstracts; over 90% of studies clearly identified the analysed concept and summarised methods and outcomes. This demonstrates researchers’ awareness of clarity and completeness in presenting their work, reflecting broader efforts to improve transparency in academic publishing (Stolt et al., 2022). However, while these sections were consistently well reported, similar rigour was not observed in later sections of the papers, suggesting an uneven application of reporting standards throughout (Lin et al., 2023).

Across the included studies, the depth and clarity of methodological and analytical reporting varied widely. Although many authors applied structured models such as Walker and Avant’s framework (Tang et al., 2024) and described their conceptual steps transparently, fewer studies incorporated quality assessment or qualitative synthesis of the included literature (McGrath et al., 2019). These omissions may stem from limited familiarity with evaluative criteria for conceptual inquiry or from misconceptions about the need for methodological rigour in theoretical work (Nor et al., 2021). Given the interpretive nature of concept analysis, underreporting of data sources and analytical procedures diminishes transparency and weakens the credibility of findings (Pollock et al., 2019). Moreover, without clear descriptions of inclusion criteria and analytic processes, readers face challenges in assessing the trustworthiness and applicability of results (Tam et al, 2019). Addressing these gaps through a discipline-specific reporting guideline will help ensure greater consistency, enhance methodological integrity and strengthen the practical contribution of concept analysis to nursing theory and evidence-based practice (Braun and Clarke, 2025).

Several key reporting items were frequently addressed, such as defining the originality of the concept (91%) and identifying its defining attributes (78%). The use of model, borderline, and contrary cases was another commonly reported feature, illustrating a commitment to exploring concepts from multiple dimensions. These techniques enrich conceptual understanding and help bridge theoretical insights with practical nursing contexts (Stevens et al., 2025). Yet, despite these strengths, the reporting of consequences, antecedents, and empirical referents while present in many studies was uneven (Pigg et al., 2025). For instance, personal consequences were described in 76% of studies, but professional and organisational consequences were often neglected, reported in only half of the cases. This uneven focus may result in incomplete conceptual models that fail to capture the full spectrum of real-world implications (Elsman et al., 2024).

One of the most underrepresented aspects of reporting pertained to ethical considerations and justification for the chosen methodology. Fewer than half of the studies explicitly discussed ethical aspects related to the conduct or implications of the concept analysis. Given that many concept analyses touch on sensitive issues such as patient autonomy, equity in care, or professional responsibilities, this omission is particularly concerning (Shamseer et al., 2015). Ethics should not be considered an optional element in conceptual scholarship but rather a foundational part of rigorous academic inquiry (Bougioukas et al., 2018). The review also revealed a notable gap in reporting practical recommendations and future research directions. Although 97% of studies summarised their findings and 88% contextualised them in discussion sections, only 61% offered concrete recommendations. Similarly, the development or inclusion of conceptual diagrams was reported in 70% of studies, suggesting that while some authors made an effort to visually synthesised their findings, others missed an opportunity to enhance understanding through graphical representation.

Regional and disciplinary patterns revealed in this review offer valuable insight into the evolving landscape of concept analysis research in nursing. The majority of studies were conducted in Asia and North America, with a predominant focus on nursing education. This trend likely reflects regional academic priorities and institutional support for pedagogical exploration. However, there is a noticeable underrepresentation of concept analysis within clinical practice, administrative and research-oriented domains, suggesting a potential gap in the broader application of conceptual inquiry in nursing (Parums,2021). Addressing this imbalance by extending conceptual investigations into these underexplored areas could foster a more comprehensive theoretical foundation and enhance the relevance of nursing science across all domains (Page and Moher, 2017). In light of these observations, the development of a structured reporting guideline specifically tailored to concept analysis in nursing becomes imperative (Foley and Davis, 2017; Helbach et al., 2023). Such a framework would resolve existing inconsistencies, elevate methodological standards and improve the reproducibility and applicability of findings. Building upon established guidelines like PRISMA (Page et al., 2021) and the Consolidated Criteria for Reporting Qualitative Research [COREQ] (Booth et al., 2014), this tailored checklist should encompass key components such as rationale for concept selection, step-by-step methodological detailing, transparency in data analysis, ethical considerations, and the use of conceptual diagrams (De Jong et al., 2021). Adopting a standardised approach of this nature has the potential to significantly enhance the quality, consistency, and scholarly impact of concept analysis research within the nursing field.

Although this review quantified reporting frequencies across key sections, a formal quality appraisal could not be conducted, as no validated tool currently exists for evaluating concept analysis studies in nursing. Consequently, while percentage trends highlight areas of reporting strength and weakness, they do not fully capture the impact of omissions on methodological rigour. Notably, inadequate reporting in the data analysis and ethical considerations sections poses the greatest threat to transparency and reproducibility, underscoring the urgent need for developing standardised quality assessment criteria for future concept analysis research.

### Bridging the reporting gap: challenges and strategies

To address the discrepancy between ideal and observed reporting practices in concept analysis, several contributing factors have been identified. The absence of discipline-specific reporting guidelines often leads to inconsistent or incomplete reporting, while concept analysis is frequently perceived as purely theoretical, resulting in underreporting of methodological details. Additionally, variations in journal expectations and peer-review standards across regions contribute to these inconsistencies. To bridge this gap, strategies such as developing and disseminating a nursing-specific reporting checklist, integrating concept analysis reporting standards into nursing education, and encouraging journal editors and reviewers to adopt the forthcoming guideline are recommended. These measures aim to enhance transparency, consistency and methodological rigour in future concept analysis publications.

### Strengths and limitations

A key strength of this review is the large number of included studies, with 111 primary concept analysis papers meeting the eligibility criteria. This substantial sample provides a robust and representative dataset for identifying consistent reporting practices. Additionally, the included studies demonstrated good methodological rigour, enhancing the reliability of the findings and ensuring that the identified reporting items are grounded in high-quality, peer-reviewed nursing research. This systematic review provides valuable insights into the reporting quality of concept analysis in nursing; however, several limitations must be acknowledged. The review included only English-language publications from peer-reviewed journals, which may have introduced language bias and excluded relevant studies published in other languages. Another important limitation lies in the subjectivity inherent to evaluating qualitative reporting items. Although data extraction was conducted by multiple reviewers and discrepancies were resolved through consensus, individual interpretation may still have influenced the assessment of reporting adequacy. Although the review aimed to reflect reporting practices across various nursing domains, the predominance of studies focused on nursing education may limit the generalisability of findings to clinical, administrative, or research settings. Despite these limitations, this review establishes a critical evidence base for guiding the development of standardised reporting guidelines, which can enhance transparency, methodological rigour, and reproducibility in future concept analysis research within the nursing discipline.

## Conclusions

This systematic review offers a thorough assessment of how concept analysis is reported within the nursing literature. Although several studies exhibited commendable strengths such as clearly defined titles, well-structured abstracts and the effective use of illustrative cases, considerable inconsistencies remain, particularly in areas related to data analysis, ethical reporting and quality assessment. These gaps point to the lack of a standardised, discipline-specific reporting framework that aligns with the distinct characteristics of concept analysis methodologies. The review’s findings emphasise the pressing need for a comprehensive guideline that incorporates core reporting elements, including the justification for concept selection, methodological transparency, ethical considerations and overall conceptual coherence. Developing such a guideline would not only improve the quality and consistency of future studies but also support the broader application of concept analysis findings in nursing education, clinical practice and policy development. This review represents a foundational step in initiating a Delphi-based process aimed at constructing a tailored reporting framework. In doing so, it sets the stage for enhancing methodological rigor and strengthening the theoretical contributions of concept analysis to nursing science. Ultimately, a standardised reporting checklist will support the global advancement of nursing knowledge and promote more impactful, reproducible scholarly work.

**Key points for policy, practice and/or research**

- Standardised reporting: The study highlights the need for standardised reporting guidelines for concept analysis in nursing, which will enhance methodological rigour and transparency across nursing research.
- Improved nursing practice: Clear and consistent reporting of concept analyses can help nurses better understand and apply theoretical concepts, improving evidence-based practice and patient care outcomes.
- Policy implications: Establishing reporting standards supports health and social care policy by promoting the use of robust and reproducible research evidence in decision-making and guideline development.
- Research advancement: The findings provide a foundational framework for future research, guiding the development of structured reporting tools and informing methodological quality in concept analysis studies.
- Educational utility: The standardised framework can also inform nursing education, supporting the teaching of theoretical and research methods to students and early-career researchers.

## Supplemental Material

sj-docx-1-jrn-10.1177_17449871251410464 – Supplemental material for Preferred reporting items for concept analysis in nursing: a systematic review Supplemental material, sj-docx-1-jrn-10.1177_17449871251410464 for Preferred reporting items for concept analysis in nursing: a systematic review by Abdulqadir J Nashwan, Jibin Kunjavara, George Joy, Kalpana Sigh, Moustaq Karim Khan Rony, Kamarudeen M and Sirwan Khalid Ahmed in Journal of Research in Nursing

sj-docx-2-jrn-10.1177_17449871251410464 – Supplemental material for Preferred reporting items for concept analysis in nursing: a systematic review Supplemental material, sj-docx-2-jrn-10.1177_17449871251410464 for Preferred reporting items for concept analysis in nursing: a systematic review by Abdulqadir J Nashwan, Jibin Kunjavara, George Joy, Kalpana Sigh, Moustaq Karim Khan Rony, Kamarudeen M and Sirwan Khalid Ahmed in Journal of Research in Nursing
