# Supplementary material for: Preferred reporting items for concept analysis in nursing: a systematic review
Source: J Res Nurs. 2026 Feb 4:17449871251410464. Online ahead of print. doi: 10.1177/17449871251410464 (PMC12872427; doi:10.1177/17449871251410464)
Supplement: sj-docx-1-jrn-10.1177_17449871251410464 – Supplemental material for Preferred reporting items for concept analysis in nursing: a systematic review [file sj-docx-1-jrn-10.1177_17449871251410464.docx]

**Process of Data Extraction and Development and Refinement**

**Step 1: Initial Extraction**

- **Reviewers:** GJ, KM, JK
- **Tool Used:** Structured spreadsheet (11)
- **Categorization:**
  - “Reported”
  - “Not Reported”
  - “Not Applicable”
- **Outcome:** Initial extraction completed for first 10 studies.

**Step 2: Identification of Gaps**

- Missing key elements specific to concept analysis reporting identified
- Need for broader and more detailed components recognized

**Step 3: Manual Re-examination**

- Each paper manually re-read by the research team
- **Actions:**
  - Added new relevant components
  - Removed duplicates
  - Refined unclear items

**Step 4: Tool Expansion**

- **New Items Added:** 56
- **Focus Areas Added:**
  - Use of reporting guidelines
  - Methodological rigor
  - Conceptual derivation
  - Clarity in analytical processes
- **Final Tool:** 66 items (reporting components + study characteristics)

**Step 5: Expert Review and Validation**

- **Expert:** AJN (Content Expert)
- **Action:** Reviewed and refined components for precision and relevance

**Step 6: Documentation and Transparency**

- All versions of the data extraction form included in **Supplementary Materials**
- Demonstrate **stepwise refinement** and ensures **transparency and reproducibility**
