# Supplementary material for: Preferred reporting items for concept analysis in nursing: a systematic review
Source: J Res Nurs. 2026 Feb 4:17449871251410464. Online ahead of print. doi: 10.1177/17449871251410464 (PMC12872427; doi:10.1177/17449871251410464)
Supplement: sj-docx-2-jrn-10.1177_17449871251410464 – Supplemental material for Preferred reporting items for concept analysis in nursing: a systematic review [file sj-docx-2-jrn-10.1177_17449871251410464.docx]

Supplementary Table: Assessment of Reporting Items in Concept Analysis Studies.

| Sl No | Reporting Item | Component | Reported(nos) | Not reported (Nos) | Not Applicable | Percentage (Reported) |
| --- | --- | --- | --- | --- | --- | --- |
| 1. | The study title explicitly defines the concept | Title &Abstract | 101 | 10 | 0 | 91 |
| 2. | The abstract clearly identifies the concept being analyzed. | Title &Abstract | 105 | 6 | 0 | 95 |
| 3. | The methodology for concept analysis is outlined in the abstract | Title &Abstract | 69 | 36 | 6 | 62 |
| 4. | The abstract includes the concept question or focus prompt. | Title &Abstract | 100 | 11 | 0 | 90 |
| 5. | The abstract details the phases of concept analysis. | Title &Abstract | 97 | 14 | 0 | 87 |
| 6. | The method used for concept analysis is explicitly described | Title &Abstract | 94 | 17 | 0 | 85 |
| 7. | Key elements of the analysis are highlighted in the abstract. | Title &Abstract | 89 | 21 | 0 | 80 |
| 8. | The results and conclusions of the analysis are clearly presented. | Title &Abstract | 107 | 4 | 0 | 96 |
| 9. | The rationale for selecting the concept analysis method is provided. | Background/ Introduction | 98 | 13 | 0 | 88 |
| 9. | The study states a clear aim or objective for the concept analysis. | Background/ Introduction | 109 | 2 | 0 | 98 |
| 10. | Justification for conducting the concept analysis is explained. | Background/ Introduction | 104 | 7 | 0 | 94 |
| 11. | The sources of literature reviewed are clearly described. | Method | 84 | 25 | 2 | 76 |
| 12. | The time frame of the included literature is mentioned. | Method | 46 | 62 | 3 | 41 |
| 13. | Keywords, search terms, and Boolean operators used are specified. | Method | 90 | 21 | 0 | 81 |
| 14. | A structured search strategy (e.g., PRISMA) is included. | Method: Framework. | 38 | 73 | 0 | 52 |
| 15. | A recognized concept analysis method (e.g., Walker & Avant) is followed. | Method: Framework. | 89 | 22 | 0 | 80 |
| 16. | The methodology is presented step by step | Method: Framework. | 105 | 6 | 0 | 95 |
| 17. | A quality assessment of selected studies is conducted. | Method: Framework. | 32 | 70 | 9 | 29 |
| 18. | A qualitative synthesis of data is provided | Method: Data Sources. | 23 | 45 | 43 | 21 |
| 19. | The types of studies included are explicitly stated. | Method:Data Sources. | 47 | 49 | 15 | 42 |
| 20. | An evidence table summarizing all the studies included is provided. | Method:Data Sources. | 53 | 47 | 11 | 48 |
| 21. | Criteria for selecting data sources are clearly defined. | Method:Data Sources. | 63 | 42 | 6 | 57 |
| 22. | Additional data collection methods (e.g., interviews, observations) are described. | Method:Data sources. | 26 | 38 | 47 | 23 |
| 23. | The process of qualitative analysis is outlined. | Data Analysis. | 56 | 39 | 26 | 50 |
| 24. | Data analysis techniques for qualitative data are mentioned. | Data Analysis. | 26 | 53 | 32 | 23 |
| 25. | The originality of the concept is discussed | Results: Define concept. | 101 | 10 | 0 | 91 |
| 26. | The purpose of the concept analysis is clearly stated. | Results: Define concept. | 71 | 40 | 0 | 64 |
| 27. | Applications of the concept in different settings are explained. | Results: Define concept | 65 | 25 | 21 | 56 |
| 28. | Surrogate terms and related concepts are identified. | Results: Define concept | 72 | 39 | 0 | 65 |
| 29. | Sub-concepts or subdomains are explored. | Results: Define concept | 47 | 61 | 3 | 42 |
| 30. | . Comparisons with similar and contrasting concepts are made. | Results: Define concept | 71 | 40 | 0 | 64 |
| 31. | Various definitions of the concept from literature are included. | Results: Define concept | 34 | 54 | 23 | 31 |
| 32. | Key characteristics and defining attributes are identified. | Results: Define concept | 83 | 28 | 0 | 78 |
| 33. | Constructed cases are clearly presented. | Results:  Define attributes | 71 | 40 | 0 | 64 |
| 34. | Real-life examples illustrating critical attributes are included. | Results:  Define attributes | 64 | 28 | 19 | 58 |
| 35. | Real example that, ideally, presents all the critical attributes. | Results: Model case | 86 | 15 | 0 | 77 |
| 36. | Exploring cases that are highly contrasting or closely resembling the concept | Results: Additional cases | 69 | 41 | 0 | 62 |
| 37. | Borderline cases are described. | Results: Additional Cases-Borderline cases. | 77 | 34 | 0 | 69 |
| 38. | Attributes that justify the concept’s definition are identified. | Results: Additional cases -Related Case | 46 | 65 | 0 | 41 |
| 39. | A contrary case is included to highlight opposing ideas. | Results: Additional cases -Contrary case | 56 | 37 | 18 | 50 |
| 40. | Invented cases are mentioned. | Results:Additional cases-**Invented Cases** | 28 | 61 | 12 | 46 |
| 41. | **Illegitimate cases are discussed.** | Results:Additional cases-**Illegitimate Cases** | 37 | 74 | 0 | 33 |
| 42. | Each case is analyzed or reflected upon. | Results: Model & additional cases | **34** | 15 | 0 | 31 |
| 43. | Antecedents (events leading to the concept) are identified. | Results:  Antecedents and consequences. | 98 | 13 | 0 | 88 |
| 44. | Situational and systematic antecedents are identified. | Results:  antecedents and consequences. | 65 | 30 | 15 | 59 |
| 45. | Cognitive, emotional, personal, and professional antecedents are discussed. | Results:  antecedents and consequences. | 74 | 35 | 0 | 67 |
| 46. | Environmental, cultural, and organizational factors are considered. | Results:  antecedents and consequences. | 61 | 51 | 0 | 55 |
| 47. | Consequences (events resulting from the concept’s occurrence) are defined. | Results:  antecedents and consequences | 64 | 35 | 12 | 58 |
| 48. | Emotional and psychological consequences are discussed. | Results:  antecedents and consequences | 51 | 60 | 0 | 46 |
| 49. | Organizational consequences mentioned. | Results:  antecedents and consequences | 65 | 46 | 0 | 59 |
| 50. | Physical consequences are mentioned. | Results:  antecedents and consequences | 58 | 53 | 0 | 52 |
| 51. | Professional consequences are included. | Results:  antecedents and consequences | 56 | 55 | 0 | 50 |
| 52. | Personal consequences included. | Results:  antecedents and consequences | 84 | 27 | 0 | 76 |
| 53. | Empirical referents (measurable indicators of the concept) are provided. | Results: Empirical referents | 96 | 15 | 0 | 86 |
| 54. | Empirical evidence supporting the concept is reported. | Results: Empirical referents | 78 | 33 | 0 | 70 |
| 55. | Hypotheses related to the concept are formulated. | Results | 18 | 93 | 0 | 16 |
| 56. | The relevance of the study findings is analyzed. | Results | 34 | 77 | 0 | 31 |
| 57. | Ethical aspects are discussed in relation to the study’s methods and results. | Ethics | 45 | 56 | 10 | 41 |
| 58. | Operational definitions based on the concept. | Operational definitions | 78 | 33 | 0 | 70 |
| 59. | Authors discussed the relevance of the  study results. | Discussion | 98 | 13 | 0 | 88 |
| 60. | A summary of findings from the study  was provided. | Discussion | 108 | 3 | 0 | 97 |
| 61. | Gaps identified and areas for future research. | Discussion | 84 | 29 | 0 | 76 |
| 62. | limitations are acknowledged and discussed. | Limitation | 87 | 24 | 0 | 78 |
| 63. | The study explores all possible applications of the concept. | Limitation | 93 | 18 | 0 | 84 |
| 64. | Nursing-related implications are explicitly mentioned. | Implications | 89 | 21 | 0 | 80 |
| 65. | Recommendations based on the findings are provided. | Recommendation | 68 | 37 | 6 | 61 |
| 66. | A conceptual diagram is included to illustrate relationships between attributes, antecedents, and consequences. | Conclusion | 78 | 31 | 2 | 70 |
